# Perturb and optimize users’ location privacy using geo-indistinguishability and location semantics

**DOI:** 10.1038/s41598-022-24893-0

**Published:** 2022-11-28

**Authors:** Yan Yan, Fei Xu, Adnan Mahmood, Zhuoyue Dong, Quan Z. Sheng

**Affiliations:** 1 School of Computer and Communication, Lanzhou University of Technology, Lanzhou, 730050 China; 2 School of Computing, Faculty of Science and Engineering, Macquarie University, Sydney, NSW 2109 Australia

**Keywords:** Energy science and technology, Mathematics and computing

## Abstract

Location-based services (LBS) are capable of providing location-based information retrieval, traffic navigation, entertainment services, emergency rescues, and several similar services primarily on the premise of the geographic location of users or mobile devices. However, in the process of introducing a new user experience, it is also easy to expose users’ specific location which can result in more private information leakage. Hence, the protection of location privacy remains one of the critical issues of the location-based services. Moreover, the areas where humans work and live have different location semantics and sensitivities according to their different social functions. Although the privacy protection of a user’s real location can be achieved by the perturbation algorithm, the attackers may employ the semantics information of the perturbed location to infer a user’s real location semantics in an attempt to spy on a user’s privacy to certain extent. In order to mitigate the above semantics inference attack, and further improve the quality of the location-based services, this paper hereby proposes a user side location perturbation and optimization algorithm based on geo-indistinguishability and location semantics. The perturbation area satisfying geo-indistinguishability is thus generated according to the planar Laplace mechanism and optimized by combining the semantics information and time characteristics of the location. The optimum perturbed location that is able to satisfy the minimum loss of location-based service quality is selected via a linear programming method, and can be employed to replace the real location of the user so as to prevent the leakage of the privacy. Experimental comparison of the actual road network and location semantics dataset manifests that the proposed method reduces approximately 37% perturbation distance in contrast to the other state-of-the-art methods, maintains considerably lower similarity of location semantics, and improves region counting query accuracy by a margin of around 40%.

## Introduction

The rapid development of mobile Internet and widespread popularization of intelligent terminals enables human beings to obtain information at anytime and from anywhere. Smartphones with positioning functions have not only become a new “organ” for humans to obtain and transmit information, but also become a natural interface between individuals and the Internet. Users can find out the surrounding traffic conditions via their smart phones, plan reasonable travel routes and implement real-time navigation, realize location-based information retrieval, query points of interest, enjoy entertainment services, or request emergency rescue, etc. Such sort of location-based value-added services are referred to as location-based services (LBS) which not only brings great convenience to the end users but also has huge commercial value attached to them^1–4^.

However, location is a kind of highly sensitive information that can reflect personal privacy. Improper collection and use of location information may lead to the disclosure of private information such as home address, living habits, health status, social relations, places of interest, and economic conditions^5,6^. Therefore, protecting location privacy of mobile terminals is the most concerned issue for users, and it’s also the most urgent task that restricts the development of location big data services.

In a traditional LBS system, it is a tacit admission that the LBS provider will not reveal the real locations of users and is trusted to handle the raw data correctly. However, in practical applications, even if the LBS provider does not actively steal or disclose users’ real location, the information stored on their server may still be leaked owing to equipment failure, communication hijacking, hacker attacks, or other issues. To address the above problems, local differential privacy (LDP)^7,8^ model is proposed to enable users to process and protect sensitive information on their respective sides and according to their personal needs. Since it is no longer necessary to provide real locations to the third-party platforms, LDP based privacy protection technology can provide users with strong guarantees of privacy and is expected to solve the privacy protection problem that restricts the development of location big data.

**Figure 1:**
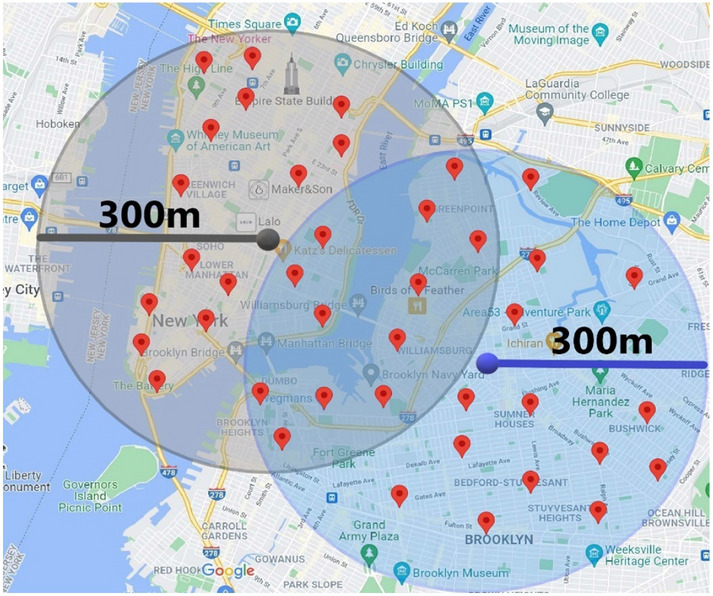
Location perturbation will lose LBS services outside the intersection area. Geo-information obtained via Google Maps (https://www.google.com/maps, Latitude: 40.7185036, Longitude: − 73.9648126, Elevation: 13.02) and the user’s quering range and POI have been marked manually.

In order to achieve location privacy protection on the user side, a natural idea is to generate and submit a fake location instead of the real one. Therefore, a perturbation mechanism is needed on the user side^9^. A good perturbation mechanism needs to face the challenges from the following aspects. First of all, the perturbation mechanism needs to balance the quality loss of location-based services and location privacy leakage. If the distance between the perturbed location and the real location is too large, the quality of location-based services will be severely compromised. As depicted in Fig. 1, the user wants to retrieve the points of interest within 300 m of his/her real location. If the perturbed location submitted by the user is far away from the real one, the results returned by the LBS server may only contains a small part of the real points of interest (POI), which will greatly reduce users’ experience of the LBS. On the contrary, if the distance between the perturbed location and the real location is too small, it may not able to prevent the leakage of location privacy and other related privacy.

Secondly, most of the existing perturbation mechanisms are designed for location information in free space^10^ and do not consider the spatiotemporal rationality of the perturbed location^11^. Perturbed locations that appear at unreasonable times and locations not only fail to protect location privacy, but may also attract the attention of attackers. For example, a user is on the coastal road to the airport, but the perturbation mechanism of free space generates a perturbation location in the sea, which is obviously unreasonable. Another example is that a user leaves the hospital where he/she works at 00:30 and is planning to take a taxi to go home. However, the location semantics of his perturbed location submitted to the LBS system is a nearby primary school. According to common sense in life, people is unlikely to have classes in school at such a time. Therefore, the attacker can naturally rule out this fake location.

In addition, the location areas with different social functions have different semantics information and sensitivity. Usually, location semantics can be classified into different categories such as medical care, education, catering, entertainment, finance, transportation, etc. If an attacker possesses the background knowledge of the road network and related location semantics, he can implement inference attack accordingly (i.e., infer users’ sensitive information such as home address, health status, and economic conditions according to their location semantics). For example, the user is reluctant to disclose his trip to the dental clinic, but the perturbed location of his destination shows that he is in the inpatient department of the hospital. Although the precise location information of the user is not exposed, the same semantics information still cannot prevent an attacker from inferring that the user has a health problem.

In order to solve the above problems, this paper proposes a local location perturbation algorithm for a single request of location-based service. The radius of the perturbation area is determined by the privacy parameter of the user side. The quality loss of LBS and the possible privacy leakage caused by location semantics are fully considered. The proposed algorithm improves the availability of perturbed location on the premise of ensuring local differential privacy protection of user’s location. The main contributions of this paper are as follows:

- A location perturbation generation algorithm is proposed based on geo-indistinguishability and location semantics which generates the optional regions for perturbed locations based on the planar Laplacian mechanism, and further optimizes the optional regions in accordance with the similarity and temporal correlation of location semantics.
- An optimal selection algorithm for the perturbed location is designed with the objective function to minimize the quality loss of the location-based service. The optimal perturbed location is selected from the optional regions by linear programming.
- Extensive experiments on real location datasets suggest that the location perturbation and optimization algorithm proposed in this paper is superior in contrast to the other existing location perturbation mechanisms in terms of privacy protection strength and data availability.

The rest of the paper is organized as follows. Section “Related work” provides an overview of relevant studies pertinent to location *K*-anonymity, geo-indistinguishability, and location semantics. Section “Prior knowledge” defines the local differential privacy and geo-indistinguishability model used in the privacy preserving data collection mechanism. Section “Proposed local perturbation and optimization algorithm” details the proposed location perturbation and optimization algorithms. Section “Experimental results” reports a set of empirical studies, whereas, section “Conclusions” concludes the paper, lays out the limitations, and future works of the research.

## Related work

In order to solve the location privacy leakage problem in LBS, various methods for environments both in free space and road network have been proposed, such as *K*-anonymity^12–18^, local differential privacy^19–27^, geo-indistinguishability^10,11,28–36^, and location semantics^37–45^.

Marco Gruteser et al. introduced the concept of *K*-anonymity in relational databases into the field of privacy protection of location-based services and proposed the location *K*-anonymity model^12^. Many studies in this category generalized users’ exact location into an area containing at least *K* users. Others replaced the initial location with a large amount of dummy locations including the real one. Gedik et al.^13^ designed a scalable architecture for location privacy protection of LBS, which includes a personalized location anonymity model and a set of location perturbation algorithms. Ni et al.^15^ constructed anonymous domains separately in dense and sparse areas. Shen proposed a location privacy protection algorithm based on a local-sensitive hashing algorithm^16^, which replaced the GPS coordinates of the user’s specific location with a set of interest points around him. Liu et al.^17^ believed that the attackers may use auxiliary information such as data analysis and crawlers to determine the approximate location of target users. Therefore, they generated virtual locations for users using the probability density function to achieve *K*-anonymity with privacy awareness in LBS. Wang et al.^18^ proposed a greedy strategy to generate secure anonymous regions based on users’ privacy requirements and real-time location. The intersection of anonymous user sets at different times is calculated and user’s identity is updated by using a dynamic pseudonym mechanism. Although *K*-anonymity is the most widely used definition of privacy for location-based systems in the literature, the main purpose of this mechanism is to protect user’s identity so that the attackers cannot infer a user amongst a set of *K* different users or make a user’s location indistinguishable amongst a set of *K* points.It may seriously degrade the location service quality and increase the query processing overhead of the server.

Allowing mobile users to perturb their locations locally before sending to the LBS provider is a promising privacy-preserving model for location collection and analysis. Kairouz et al.^19^ designed a binary response mechanism and a random response mechanism for local differential privacy and applied them for location privacy protection. The private spatial data aggregation method proposed by Chen et al.^20^ presents a novel framework that allows an untrusted server to accurately learn the users’ distribution over a spatial domain while satisfying personalized local differential privacy for each user. Dai et al.^21^ proposed a privacy preserving framework for worker’s location in spatial crowdsourcing based on LDP model. The noisy locations of workers are submitted to the spatial crowdsourcing server rather than the real locations. Alvim et al.^22^ proposed a local differential privacy geometric mechanism for location data. The local differential privacy exponent mechanism proposed by Gursoy et al.^23^ can provide better statistical utility while preserving location privacy. Zhao et al.^24^ proposed a probabilistic top-down partitioning algorithm to generate location-record data under local differential privacy which employs a carefully designed partition tree model to extract essential information in terms of location records and maintains high utility while providing privacy guarantees. Hong et al.^25^ investigated the problem of collecting locations of individual users under LDP and proposed the square mechanism to collect the geospatial data by reducing the MSE of each location. Sun et al.^26^ used LDP for distance estimation between distributed data. The LDP-based location collection and protection methods prevent the location privacy of users from being compromised by data collectors and potential attackers. Compared with the centralized differential privacy model, the LDP-based location collection methods provide strong guarantees of privacy. However, when the aggregator attempts to infer the data distribution based on the randomized information sent by a lot of users, the LDP-based methods produces more statistical errors than the DP-based methods^27^.

Andres et al. proposed the concept of geo-indistinguishability^10^ for the privacy protection of location-based systems. This mechanism introduced controlled noise to the user’s exact location to obtain an approximate location and then sent it to the LBS provider in order to obtain desired service. Within a circular region of radius *r*, the attacker can barely tell the difference between the approximate location and the real location. Chatzikokolakis et al.^28^ proposed two approaches to achieve geo-indistinguishability for generic locations and custom locations respectively, and extended the proposed mechanism to the case of location tracking. Hua et al.^29^ partitioned the planar location area into several hexagons and combined the geo-indistinguishability to reduce the loss of privacy parameters by publishing the location of the centroid of each hexagon. Takagi et al.^11^ proposed the geo-graph-indistinguishability privacy protection mechanism based on the road network environment, which takes the road intersection as the perturbed location of user and improves the shortcomings of the geo-indistinguishability mechanism in the privacy and utility of the actual road network. Qiu et al.^30^ applied geo-indistinguishability to solve the problem of vehicle-based spatial crowdsourcing location privacy protection on road networks, and designed a location obfuscation strategy to reduce the quality loss caused by obfuscation. Arain et al.^31^ proposes an algorithm to protect the information of mobile vehicle’s users and use geo-indistinguishability to obtain a set of POIs near the source location and destination location. Luo et al.^32^ first classified the location set through a density-based clustering algorithm and then perturbed the real locations according to geo-indistinguishability so as to solve the problem of privacy leakage caused by frequent check-in. Xiong et al.^33^ applied geo-indistinguishability to spatial crowdsourcing and combined location obfuscation and path optimization to provided strong privacy protection with minimal cost. Al-Dhubhani et al.^34^ investigated the potential correlations between obfuscated locations generated according to geo-indistinguishability in continuous query services.The location perturbation mechanism based on geo-indistinguishability releases approximate locations to obtain corresponding location services. Therefore, the quality of location-based service obtained by users varies with the fluctuation of the distance between the disturbed location and the real location. In addition, not reporting the real location of a user does not mean that the user’s location cannot be inferred. Actually, it could be inferred by the prior knowledge or side information obtained by the attackers^35^.

The contextual information attached to the location data exposes more private information of the users. The effect of location anonymity and perturbation will seriously decline if the attackers have obtained this kind of contextual information (i.e., location semantics). Therefore, many location privacy protection methods incorporate location semantics to enhance their protection effect. Xiao et al.^37^ analyzed the problem that location *K*-anonymity suffers from homogeneity attacks due to the lack of location semantics diversity. They proposed a *p*-sensitive privacy-preserving model to realize location anonymity while considering query diversity and location semantics. Lee et al.^38^ suggested to learn semantics information from location data and let trusted anonymity servers perform location anonymization by hiding semantically heterogeneous locations. Berker et al.^39^ introduced an inference model considering location semantics and privacy-preserving mechanisms, and conducted a formal analysis of the bidirectional problem between semantics level and location inference. The PrivSem privacy protection framework proposed by Li et al.^40^ integrates location *k*-anonymity, segmental-semantics diversity, and differential privacy to protect user’s location privacy from infringing. Wang et al.^41^ suggested to calculate the semantics distance and query probability between fake locations and build a location semantics tree to satisfy the diversity. In Kuang et al.^42^, the sensitive weight document is automatically generated according to the user’s sensitivity to the semantics of different locations. Then, the *K*-anonymous optimal cooperative segment of the user’s location is obtained through the reinforcement learning algorithm. Finally, user’s location and query location have been perturbed based on the location semantics of the real road network environment. Bostanipour et al.^43^ proposed a joint obfuscation algorithm based on mixing semantics label to solve the problem of privacy leakage that may occur in anonymous regions. Min et al.^44^ designed a location perturbation strategy based on reinforcement learning, which adaptively selects perturbation strategy according to the sensitivity of location semantics. However, the current location protection schemes which combined with semantics do not have the unified classification of location semantics. Different research schemes adopt their own designed or defined semantic classification trees which makes it difficult to compare the performance of the different methods. Besides, most of the existing methods only employ certain types of location semantics. Whether there are other available types of location semantic information and whether a specific type of semantic information is more important than the other is a worth investigation phenomenon.

## Prior knowledge

In order to facilitate the understanding of subsequent definitions and descriptions, we provide a unified explanation of the mathematical notations defined and employed in this paper (as depicted in Table 1).

**Table 1:** Mathematical notations.

Symbol	Description
$\varepsilon$	Privacy parameter
$d(x_{1},x_{2})$	The distance between any two locations $x_{1}$ and $x_{2}$
$x_{0}$	User’s real location
$x^{'}$	The perturbed location
$f_{\varepsilon }(r,\theta )$	The probability density function in polar coordinates
$P_{area}$	Perturbation Area
$LS_{matrix}$	Location semantic matrix
$Cos(D_{a},D_{b})$	The cosine similarity between two semantic location $D_{a}$ and $D_{b}$
$\rho$	The lower limitation of the number of users
$O_{area}$	Optimized area
$QL(K,\pi ,d)$	Quality loss of the LBS services (with perturbation matrix *K*, prior probability $\pi$, and distance *d* between the real location and the perturbed location
$M_{dis}$	The mean value of the distance between the real location and the perturbed location
$V_{dis}$	The variance of the distance between the real location and the perturbed location
*RE*(*Q*)	Relative error within querying range *Q*

### Local differential privacy

**Figure 2:**
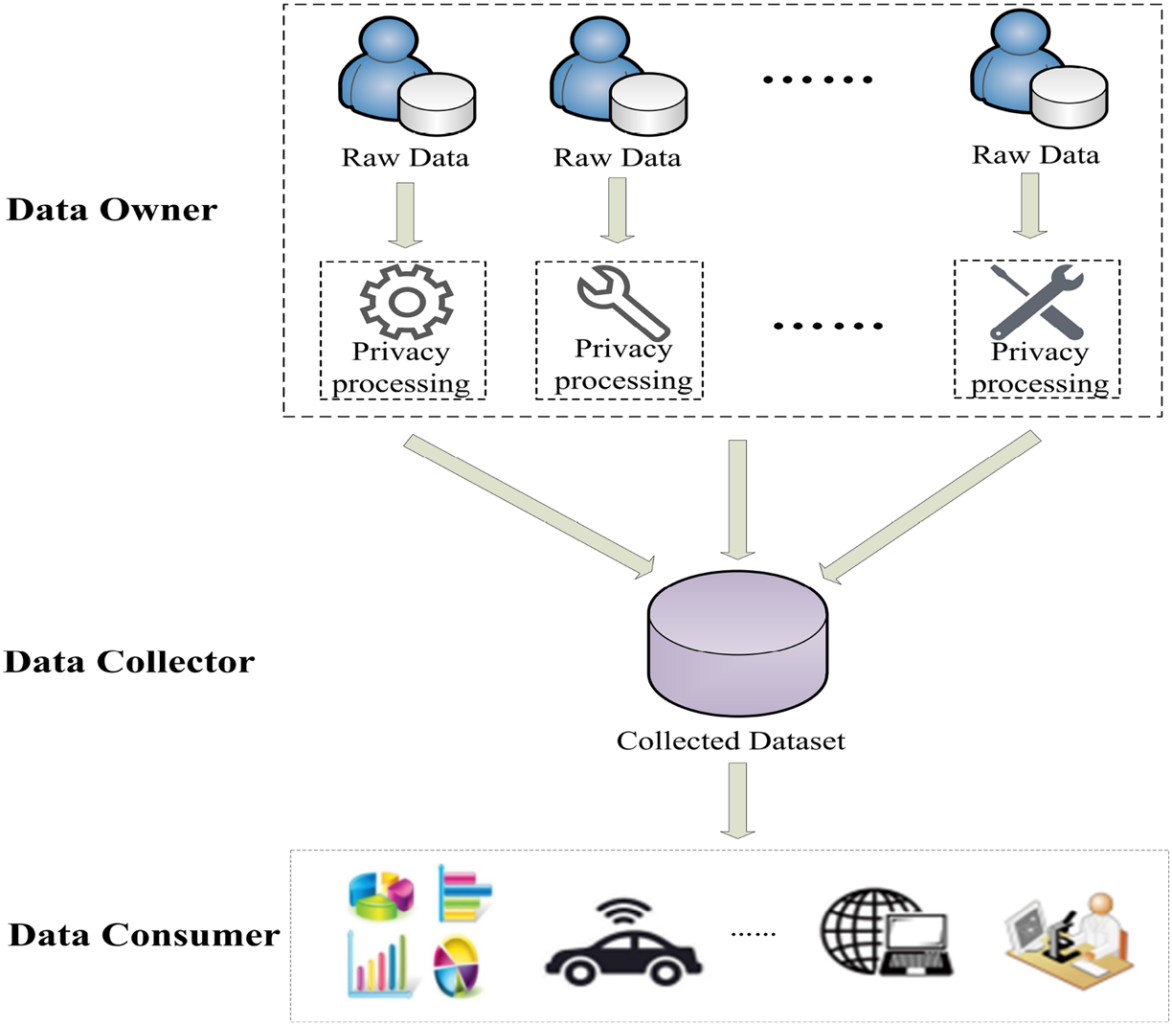
Data processing framework based on LDP.

Traditional data collection process adopts an honest model, which default the data collection platform will not actively steal or leak the sensitive information of users. However, in practical applications, even if the data collection platform does not collect or illegally use users’ sensitive information, attackers still can steal or destroy data through system vulnerabilities. In order to avoid user privacy leakage caused by untrusted third-party data collection platforms, the local differential privacy model (LDP)^7,8^ is proposed. This model fully considers the background knowledge of any attacker and quantifies the degree of privacy protection. Each of the data owner can implement privacy processing on their own data independently and then send the data to the collector (as depicted in Fig. 2). The centralized data privacy protection process originally undertaken by the data collection platform is pre-transferred to each data owner, enabling them to process and protect personal sensitive information individually, and perform more personalized privacy protection. Therefore, the intervention of trusted party is no longer required and privacy attacks that may be caused by untrusted third-party data collectors are also avoided.

#### Definition 1

^7,8^ An algorithm *A* satisfies $\varepsilon$-local differential privacy if and only if for any input $x_{1}$ and $x_{2}$ there is:

1 $$\begin{aligned} \forall y \in Range(A): Pr[A(x_{1})=y]\le e^{\varepsilon }\cdot Pr[A(x_{2})=y] \end{aligned}$$

where *Range*(*A*) denotes the set of all possible outputs of algorithm *A*. The privacy parameter $\varepsilon \ge 0$ represents the privacy protection strength. The smaller value of $\varepsilon$ can provide higher privacy protection strength.

### Geo-indistinguishability

For the location privacy protection on user side, most of the traditional methods generalize a user’s precise location into a location area including other nearby users, or send a large amount of fake locations together with the real one to protect it. The result of this solution not only increases communication and data transmission overhead but also severely degrades the quality of location-based services. To address the above issues, Andres et al. proposed the concept of geo-indistinguishability^10^. This mechanism incorporates controlled noise to the user’s real location to obtain an approximate location. Within a circular area of radius *r*, the attacker can hardly tell the difference between the perturbed and the real locations.

#### Definition 2

^10^ For a finite Euclidean space $\chi$, a mechanism *A* satisfies $\varepsilon$-geo-indistinguishability if for all $x_{1}, x_{2} \in \chi$, $Z \subseteq {\mathbb {Z}}$, there is:

2 $$\begin{aligned} A(x_{1})(Z)\le e^{\varepsilon \cdot d(x_{1},x_{2})}\cdot A(x_{2})(Z) \end{aligned}$$

The definition of geo-indistinguishability allows a user to disclose enough location information in order to obtain the desired service. In Eq. (2), *d*(.) stands for the distance metric. In a real physical environment, it can be represented by the Euclidean distance between the two location points. In fact, geo-indistinguishability is an instance of a generalized variant of local differential privacy with a distance metric. Comparing Eqs. (1) and (2), we can observe that when $d(x_{1},x_{2} )=1$, geo-indistinguishability is equal to local differential privacy.

#### Definition 3

^10^ Given the privacy parameter $\varepsilon \in {\mathbb {R}}^{+}$ and the actual location $x_0 \in {\mathbb {R}}^{2}$, the probability density function of the planar Laplacian centered at $x_0$ can be expressed as:

3 $$\begin{aligned} f_{\varepsilon }(x_0)(x^{'})=\frac{\varepsilon ^{2}}{2\pi }\cdot e^{-\varepsilon \cdot d(x_0,x^{'})} \end{aligned}$$

where $\frac{\varepsilon ^{2}}{2\pi }$ is a normalization factor.

### Location-based services

**Figure 3:**
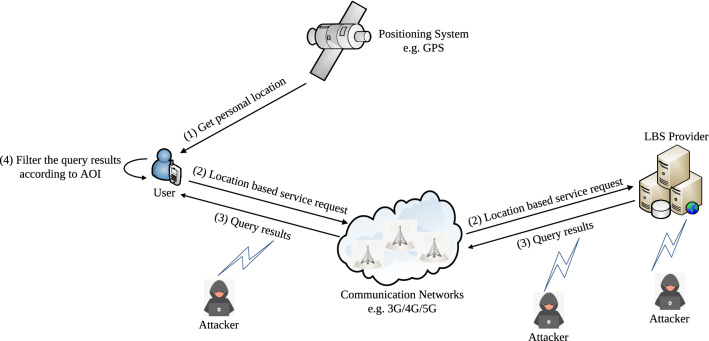
A simple framework of the LBS system.

A simple framework of the LBS system is portrayed in Fig. 3 which uses positioning technology to acquire location movements of mobile users or terminals. The most outstanding example of such a positioning system is the GPS. With the support of the geographic information system (GIS), the LBS provider can supply various types of value-added services such as vehicle navigation, POI search, and location sharing. Communication networks provide the transmission medium for information exchange between the users and the LBS providers.

The process of obtaining the LBS mainly includes the following steps. The users firstly ascertain their precise location coordinates via their respective positioning system (i.e., it is generally considered that the location information provided by the positioning system is timely and accurate) and then initiate a location-based query request to the LBS provider together with their requirements. The LBS provider retrieves the points of interest for the users according to their submitted locations and feeds back the area of request (AOR). Finally, the user filters the query results according to his/her area of interest (AOI).

Although LBS offer remarkable convenience to the end users, it also present potential privacy risks at the same time. Users are required to submit their exact locations to receive accurate service support. Attackers may eavesdrop or steal data through the wireless communication environment or even from the LBS system (as depicted in Fig. 3) and, therefore, obtain detailed information, including but not limited to users’ current locations, points of interest, and service requirements. Adversaries with adequate accessibility to users’ data may use the location information for some particular motives and may also link the location with other publicly available data to infer privacy information of the users.

## Proposed local perturbation and optimization algorithm

The local differential privacy model provides a theoretical basis for decentralized location data collection. Users can independently perform privacy processing on their own location data according to different privacy protection requirements, and obtain various services based on the location after privacy protection processing. The local perturbation and optimization algorithm proposed in this paper firstly generates a perturbed location area that conforms to geo-indistinguishability according to the plane Laplace mechanism and user’s privacy parameter. Then, the area of perturbed location is optimized by combining the location semantic information and temporal relationships. Finally, the optimal perturbed location is selected from the remaining perturbed location area by the linear programming method and with the objective function of minimizing LBS quality loss. The proposed method can (a) provide location privacy protection for the end user during a single request of LBS and (b) maintain better service quality.

### Generate perturbation area based on geo-indistinguishability

The traditional localized differential privacy model (LDP) realizes the privacy protection of a user’s data through random response mechanism. When a user’s data consists of multiple parameters, the random response mechanism can be applied on each kind of parameter. However, this approach ignores the association between the parameters. Especially, the location information, the longitude information, or the latitude information cannot be analyzed in isolation as this would seriously damage the usability of the original location information.

Geo-indistinguishability can be seen as a generalized form of LDP, which is an extension of the differential privacy model in the 2D space. The definition of geo-indistinguishability (i.e., Definition 2) introduces a distance metric to the concept of local differential privacy. Algorithm satisfying geo-indistinguishability can return a perturbed location closer to the real location with a larger probability and a perturbed location farther from the real location with a smaller probability.Therefore, it is particularly suitable for localized differential privacy protection of location information. According to Eq. (2), the attacker can hardly tell the difference between the perturbed and the real locations within a circular area (which is controlled by the privacy parameter $\varepsilon$).

The frequency oracle^46,47^ for enabling the estimation of the frequency of location in area *D* can be specified as follows:

4 $$\begin{aligned} {\forall x^{'} \in D: Pr[A(x_{0})=x^{'})] = \left\{ \begin{array}{ll} \frac{e^{\varepsilon }}{e^{\varepsilon }+|D|-1}, &{}\quad if\quad x^{'} = x_{0} \\ \frac{1}{e^{\varepsilon }+|D|-1}, &{}\quad if\quad x^{'} \ne x_{0} \\ \end{array} \right. } \end{aligned}$$

where, $x_{0}$ and $x^{'}$ represent the real location and the perturbed location respectively and |*D*| stands for the number of perturbed locations. This kind of random response protocol sample the real location with higher probability and all the other perturbed locations with lower uniform probability.

In order to facilitate the use of the plane Laplace mechanism^10^ to achieve geo-indistinguishability, the probability density function of the plane Laplace mechanism is converted into the probability density function in polar coordinates:

5 $$\begin{aligned} f_{\varepsilon }(r,\theta )=\frac{\varepsilon ^{2}}{2\pi }re^{-\varepsilon r} \end{aligned}$$

wherein, *r* represents the distance between the initial location $x_0$ and the perturbed location $x^{'}$, and $\theta$ is the angle formed by the line $x_0 x^{'}$ with the horizontal axis of the Cartesian system.

The two random variables representing radius and angle are independent, therefore, the probability density function of the planar Laplace mechanism in polar coordinates^10^ can be expressed as:

6 $$\begin{aligned}{} & {} f_{\varepsilon }(r,\theta )=f_{\varepsilon ,R}(r)f_{\varepsilon ,\Theta }(\theta ) \end{aligned}$$

7 $$\begin{aligned}{} & {} f_{\varepsilon ,R}(r)=\int ^{2\pi }_{0}f_{\varepsilon }(r,\theta )\textrm{d}\theta =\varepsilon ^{2}re^{-\varepsilon r} \end{aligned}$$

8 $$\begin{aligned}{} & {} f_{\varepsilon ,\Theta }(\theta )=\int ^{\infty }_{0}f_{\varepsilon }(r,\theta )\textrm{d}r=\frac{1}{2\pi } \end{aligned}$$

According to the plane Laplace mechanism in the polar coordinates mentioned above, the user’s real location $x_0$ can be perturbed into a fake one $x^{'}$ that satisfies geo-indistinguishability. In order to reduce the influence of the selection of the two random variables of radius and angle on the perturbed location, the average distance can be calculated by multiple iterations and used to represent the distance $d(x_0,x^{'})$ between the perturbed location and the real location.

#### Definition 4

Let the user’s real location $x_0$ be the center of the circle, and the average distance generated by the plane Laplace mechanism be the radius, all the geo-indistinguished locations that satisfy user’s privacy requirement $\varepsilon$ constitute a perturbation area:

9 $$\begin{aligned} P_{area}=\left\{ center = x_{0},radius = \frac{1}{N}\times \sum _{i=1}^{N}r_{i}\right\} \end{aligned}$$

wherein *N* is the number of geo-indistinguished locations in the perturbation area.

**Figure 4:**
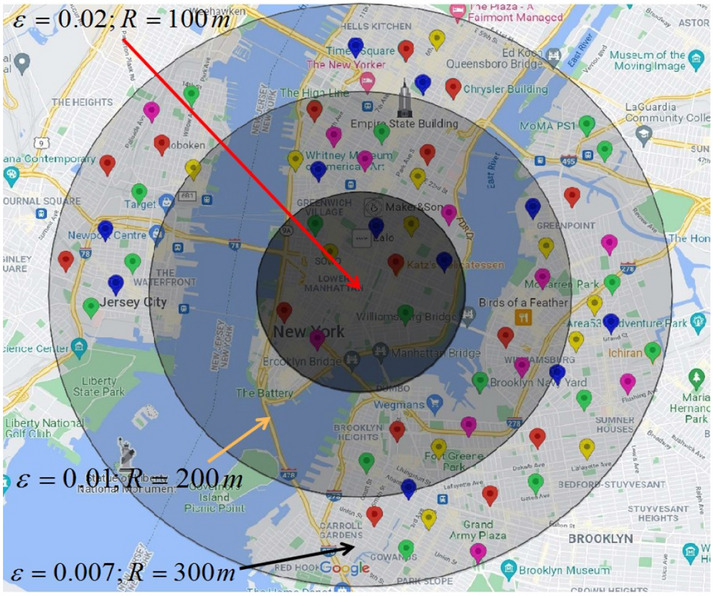
Variations of perturbation area with privacy parameter $\varepsilon$. Geo-information obtained via Google Maps (https://www.google.com/maps, Latitude: 40.7185036, Longitude: − 73.9648126, Elevation: 13.02) and POI with different semantic information have been marked manually with different colors.

Algorithm 1 depicts the pseudocode of the perturbation area generation algorithm. Lines 3–5 generate the perturbation area according to the plane Laplace mechanism using the Lambert function *W* (the − 1 branch)^10^. Line 6 generates the perturbed location relative to a user’s real position. Considering the randomness of the disturbance generated by the Laplace mechanism, line 9 calculates the average disturbance distance for all the iterations and set the result as the radius of the perturbation area. Figure 4 portrays the perturbation areas corresponding to different privacy parameters $\varepsilon$. As the decrease of the privacy parameter $\varepsilon$, the perturbation introduced by the planar Laplace mechanism becomes larger, and the coverage of the generated perturbed area is also larger.
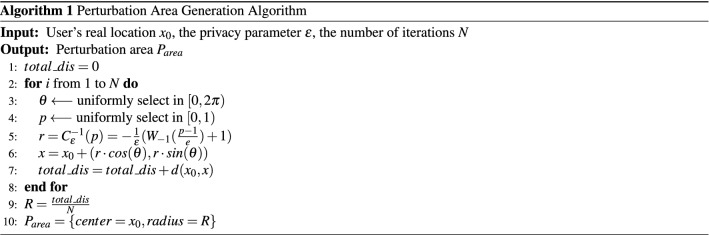

### Optimize perturbation area based on location semantics

Different geographic areas in a city provide different services and play different social roles for users, which is called the semantic information of location. The user’s appearance frequency and dwell time in different geographical areas portrays the degree of association between the user and the semantic information of the location, and then reflect the user’s living habits and behavior patterns. Attackers can infer the user’s private information based on the semantic information of his/her location, which is called the semantic inference attack. For different users, the semantic sensitivity of different locations is different, therefore, the impact of privacy leakage caused by semantic inference attacks is also different. For example, for doctors and nurses working in hospitals, the leakage of location information on the workplace will not have too much impact on them. They may be more concerned about the privacy of their home addresses. While for ordinary users, they might be more worried about the leakage of their location information when they in the hospital, which will lead to semantic inference attack on the privacy of their health status. In addition, the statistical properties of location semantics are closely related to time. The distribution characteristics of different location semantics are variant during the same time period. There are also very obvious changes in the statistical properties of the same semantic location at different times. For example, as the main place for entertainment at night, a bar always have more customers at night but few customers at working time during the day. In contrast, semantic locations such as banks, transportation hubs, schools, etc. always have more people at working time than at leisure time.

Most of the privacy protection algorithms based on location semantics combine location semantics with the *K*-anonymity model to achieve semantic diversity and improve location privacy protection effect. However, the existing location privacy protection algorithms based on semantic information do not explicitly propose a standard definition of location semantics and a method for distinguishing different location semantics. To avoid significant bandwidth overhead that users may encounter as a result of real time data download when using location-based services, we select historical location data and corresponding semantic information to set up the time series representation for location semantics. Although the historical location data may not depict the current state of a city, it can to a certain extent reflect the population distributions of different semantic locations in the city and the importance of changes over time. In this section, different types of location semantics in the same city and during the same period of time are selected to implement the statistical analysis. A perturbed area optimization algorithm based on location semantics is proposed which facilitate eliminating unreasonable locations in the perturbed area and enhancing the effect of local location privacy protection.

#### Definition 5

Let the vector $D_{i}=[N_{i1},\ldots ,N_{ij},\ldots ,N_{it}]^{'}$ be the statistical information of the *i*th location semantics at different time parameters, where $N_{ij}$ is the number of people who appear in the *i*th location semantic region during the *j*th time period. Therefore, the location semantic matrix of a city can be expressed as:

10 $$\begin{aligned} LS_{matrix} =[D_{1},D_{2},\ldots ,D_{m}] = { \left[ \begin{array}{cccc} N_{11} &{}\quad N_{21} &{}\quad \cdots &{}\quad N_{m1} \\ N_{12} &{}\quad N_{22} &{}\quad \cdots &{}\quad N_{m2} \\ \vdots &{}\quad \vdots &{}\quad \ddots &{}\quad \vdots \\ N_{1t} &{}\quad N_{2t} &{} \quad \cdots &{}\quad N_{mt} \\ \end{array} \right] } \end{aligned}  $$

wherein, *m* represents the number of location semantic types in the city.

When the location semantics can be expressed in the form of vector, the cosine similarity can be used to measure the similarity^45^ between two location semantics. The smaller the angle between the two vectors, the higher the similarity between them. Therefore, if the cosine similarity value between two location semantics is closer to 1, it means that the similarity between the two semantic locations is higher (Eq. (11)).

11 $$\begin{aligned} Cos(D_{a},D_{b})=\frac{\vec {D_{a}}\cdot \vec {D_{b}}}{\left| \vec {D_{a}}\right| \cdot \left| \vec {D_{b}}\right| } \end{aligned}$$

Considering that the real location where the user submits his location-based service request also has location semantic information, if we simply select one of the perturbed location from the perturbed area generated by Algorithm 1 to replace the user’s real location, it is very likely that the perturbed location and the real location belong to the same semantic type or have higher similarity. In order to prevent attackers from inferring users’ location privacy based on semantic information in the road network and prior knowledge of users’ distribution, we propose a perturbation area optimization algorithm based on location semantics. Let $N_t(x)$ be the number of people at a location *x* at time *t* and the lower limitation of the number of users be $\rho$. The proposed perturbation area optimization algorithm mainly has two stages: Firstly, it will delete those perturbed locations where the number of users is less than the lower limitation of the number of users, which is easy to reveal the presence of users due to the lack of group masking effect. Secondly, it will remove those perturbed locations whose semantic similarity is higher than the average similarity. Since these locations have highly semantic similarity with the user’s real location, it is easy for attackers to infer other privacy by virtue of location semantic features. Let *N* be the number of locations within the perturbation area $P_{area}$, the average semantic similarity can be expressed as Eq. (12):

12 $$\begin{aligned} &{\overline{COS}} = \frac{1}{N}\times \sum _{i=1}^{N}Cos(D_{x_0},D_{x_i})&x_{0},x_{i}\in P_{area} \end{aligned}$$

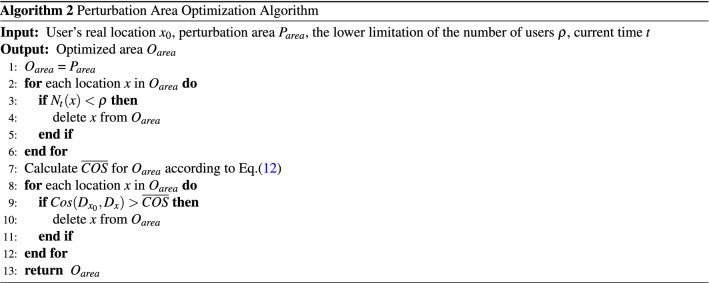


Algorithm 2 portrays the pseudocode of the perturbation area optimization algorithm based on location semantics. Line 1 assigns all the perturbed locations in $P_{area}$ to the optimized area $O_{area}$. Lines 2–6 filter out the perturbed locations with the number of users less than the lower limitation $\rho$. Line 7 calculates the average semantic similarity for the remaining locations in $O_{area}$. Lines 8–12 delete the locations with higher semantic similarity than the average value. Therefore, the rest locations in $O_{area}$ have lower semantic similarity but more number of people which facilitate improving the privacy protection effect of perturbed location and reducing the selection range of the optimal perturbed location.

**Figure 5:**
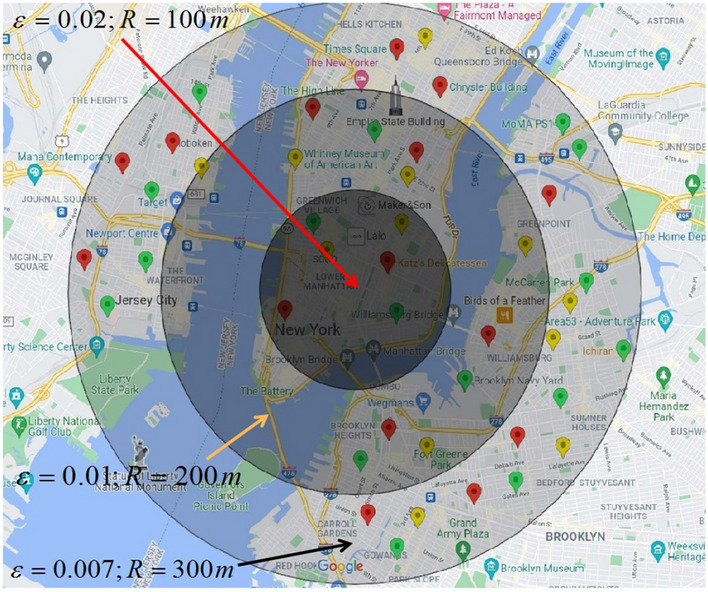
The optimized effect of perturbation region on the basis of Fig. 4. Geo-information obtained via Google Maps (https://www.google.com/maps, Latitude: 40.7185036, Longitude: − 73.9648126, Elevation: 13.02) and POI with different semantic information have been marked manually with different colors.

Figure 5 is the optimization result of the perturbed area obtained from Fig. 4. According to the proposed definition of location semantic matrix, Algorithm 2 further eliminates the disturbed locations that have over threshold value of semantic similarity with the user’s real location and do not meet the lower limitation of the number of users on the basis of Algorithm 1.

### Optimal selection algorithm based on linear programming

As mentioned above, the attackers may collect and obtain the semantic information of the road network and the prior knowledge of users’ distribution by different ways. These background knowledge may help the attackers to infer users’ location privacy. Let’s consider the following scenario: there are 4 locations A, B, C, and D, and the attackers know that the number of people in A, B, C and D is 10, 20, 30, and 40 respectively based on prior knowledge. Therefore, it can be considered that the prior probabilities of the users’ real location in the above four locations are $\pi _A=0.1$, $\pi _B=0.2$, $\pi _C=0.3$, and $\pi _D=0.4$. So the attackers may infer that the user is in location *D* at the current time with a probability of $40\%$. Combining this phenomenon, it is easy to obvious that although Algorithm 2 has optimized the perturbed area with location semantics and has reduced the leakage of location semantic information, the problem of prior probability inference is still exists. Therefore, this section proposes an optimal selection algorithm for the perturbed locations based on Algorithm 2.

#### Definition 6

The prior probability of location *x* within area $\chi$ at time *t* can be expressed by the ratio of the number of people at a location *x* to the total number of people at all locations in $\chi$.

13 $$\begin{aligned} \pi _{x}=\frac{N_t(x)}{|\chi |_t} \end{aligned}$$

wherein, $N_t(x)$ represents the number of people at location *x* at time *t* and $|\chi |_t$ manifests the total number of people at all locations in $\chi$ at the same time.

#### Definition 7

^36^ For arbitrary location *x* within the perturbation area, the service quality loss caused by the location privacy protection mechanism can be expressed as:

14 $$\begin{aligned} QL(K,\pi ,d)=\sum \limits _{x,x^{'}}\pi _{x}k_{x,x^{'}}d(x,x^{'}) \end{aligned} $$

wherein, $\pi _{x}$ is the prior probability that the user is located at *x*, *K* is the perturbation matrix, $k_{x,x^{'}}$ stands for the probability of perturbation from location *x* to location $x^{'}$, and $d(x,x^{'})$ represents the Euclidean distance from *x* to $x^{'}$.

In order to improve the LBS service quality obtained based on the perturbed location, the optimal selection algorithm proposed in this section constructs a linear programming function with the objective of minimizing the loss of service quality:

15 $$\begin{aligned}{} & {} Minimize:QL(K,\pi ,d) \end{aligned}$$

16 $$\begin{aligned}{} & {} Subject\ to \left\{ \begin{array}{ll} k_{x,z}\le e^{\varepsilon d(x,x^{'})}k_{x^{'},z}, &{}\quad \forall x,x^{'},z\in \chi \\ k_{x,z}\ge 0, &{}\quad \forall x,z\in \chi \\ \sum _{z\in \chi }k_{x,z}=1, &{}\quad \forall x\in \chi \\ \end{array} \right. \end{aligned}$$

The parameter $\chi$ used in the constraint conditions represents the set of all the locations in the finite space, $x, x^{'}, z\in \chi$. The constraint conditions contain three aspects: firstly, the perturbed locations must satisfy geo-indistinguishability; secondly, the perturbation probability must be larger than 0; finally, the sum of all the perturbed location probabilities with respect to the real location *x* must be 1.

If the optimized area contains *n* candidates, the linear programming function in Eq. (16) will receive a perturbation matrix $K_{n \times n}$ as shown in Eq. (17). Each of the element $k_{x_{i}x_{j}}$ in the perturbation matrix stands for the probability of perturbation from location $x_i$ to location $x_j$.

17 $$\begin{aligned} K_{n \times n} = { \left[ \begin{array}{cccc} k_{x_{0}x_{0}} &{}\quad k_{x_{0}x_{1}} &{}\quad \cdots &{}\quad k_{x_{0}x_{n-1}} \\ k_{x_{1}x_{0}} &{}\quad k_{x_{1}x_{1}} &{}\quad \cdots &{}\quad k_{x_{1}x_{n-1}} \\ \vdots &{} \quad \vdots &{}\quad \ddots &{} \quad \vdots \\ k_{x_{n-1}x_{0}} &{}\quad k_{x_{n-1}x_{1}} &{}\quad \cdots &{}\quad k_{x_{n-1}x_{n-1}} \\ \end{array} \right] } \end{aligned} $$

It should be noticed that there is a certain probability to return the user’s real location according to the perturbation matrix $K_{n \times n}$. To a certain extent, this is determined by the privacy parameter $\varepsilon$. When the value of the privacy parameter $\varepsilon$ is large, the error introduced by the Laplace mechanism is small, and the perturbed location is likely to return the user’s original true location. In order to prevent this from happening, the value corresponding to user’s real location in the row vector can be removed, and an optimal perturbed location can be returned according to other remaining probability values.
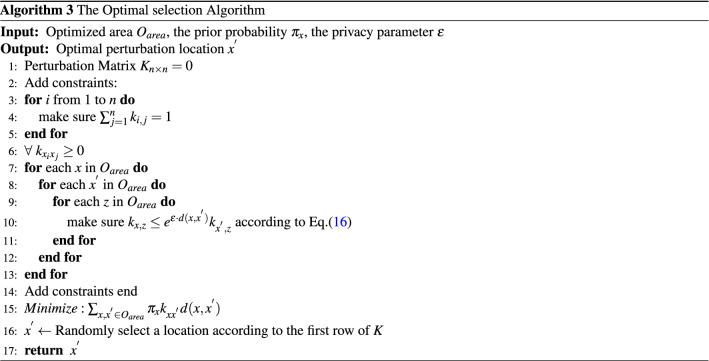


Algorithm 3 portrays the pseudocode of the optimal selection algorithm which consists of two stages. The first stage (i.e., lines 2–14) incorporates the constraints mentioned in Eq. (16). Among them the first one (i.e., lines 3–5) requires that the sum of each row in the perturbation matrix *K* must be 1 implying that the sum of the probabilities of perturbing the original location $x_{0}$ to all the other possible locations must be 1. For the optimized area $O_{area}$ with *n* candidate locations, this process needs to calculate all the elements within the matrix *K* and, therefore, the computational complexity for this part is $O(n^{2})$. The second constraint (i.e., line 6) mandates that each of the element within the perturbation matrix *K* must be greater than 0. The computational complexity of this part is *O*(*n*). The third constraint (i.e., lines 7–13) ensures that the perturbed locations meet the requirement of geo-indistinguishability (as defined in Eq. (2)). To achieve this purpose, three nested loops are required. Therefore, the computational complexity of this part is $O(n^{3})$. The second stage of the proposed algorithm (i.e. line 15) solves the linear programming problem according to the minimization objective function. In this paper, we use *Gurobi*^48^ to solve the linear programming problems which uses the primal simplex method to solve the linear programming problem with the exponential time complexity. Concurrent optimizers in *Gurobi* run multiple solvers on multiple threads simultaneously and choose the one that finishes first.

### Privacy analysis

The envisaged location perturbation and optimization algorithm based on geo-indistinguishability and semantic aims at scenarios of requesting LBS services on locations with semantic information in the road network, which is very consistent with the applications of location-based big data in our real life. Suppose an attacker has obtained the following background knowledge:

- The attacker has the road network information of the city including the distribution of various semantic locations;
- The attacker can obtain the number of users at any time and in any area that he needs, but cannot identify a specific user from it;
- The attacker may capture the information submitted to or returned back from the LBS platform.

The following will prove that the proposed location perturbation and optimization algorithm can provide $\varepsilon$-geo-indistinguished local differential privacy protection for a user’s location and resist the semantic related inference attack at the same time.

#### Proof

Our proposed solution consists of three algorithms. Firstly, the perturbation area $P_{area}$ will be generated by using Algorithm 1 according to a user’s real location $x_0$ and privacy parameter $\varepsilon$. Then, the perturbation area $P_{area}$ will be optimized via Algorithm 2 based on the similarity and temporal correlation of location semantics. Finally, the optimal perturbed location will be selected via Algorithm 3 by using a linear programming function. Therefore, to prove that the output perturbation location of the proposed algorithm satisfies $\varepsilon$ geo-indistinguishability, it is only necessary to prove that all the locations within the perturbation area $P_{area}$ generated by Algorithm 1 conform to $\varepsilon$ geo-indistinguishbility.

Let $x_{0}$ be the real location of a user, $x^{'}$ depicts the perturbed location generated according to the plane Laplace mechanism, and the distance between real location and perturbed location corresponds to the radius of the perturbation area $P_{area}$. Let $x_{i}$ be one of the arbitrary location within $P_{area}$, therefore, we only need to prove that $x_{i}$ satisfies $\varepsilon$ geo-indiscernibility. According to reference [10], it can be implied that the plane Laplace mechanism conforms to $\varepsilon$ geo-indiscernibility. Let *PL* represent the plane Laplace mechanism. Accordingly,

$$\begin{aligned} Pr[PL(x_{0})=x^{'}]\le e^{\varepsilon \cdot d(x_{0},x^{'})}\cdot Pr[PL(x^{'})=x^{'}] \end{aligned}$$

and

$$\begin{aligned} Pr[PL(x_{0})=x_{i}]\le e^{\varepsilon \cdot d(x_{0},x_{i})}\cdot Pr[PL(x_{i})=x_{i}] \end{aligned}$$

so that:

$$\begin{aligned} \frac{Pr[PL(x_{0})=x^{'}]}{Pr[PL(x_{0})=x_{i}]}&\le \frac{e^{\varepsilon \cdot d(x_{0},x^{'})}\cdot Pr[PL(x^{'})=x^{'}]}{e^{\varepsilon \cdot d(x_{0},x_{i})}\cdot Pr[PL(x_{i})=x_{i}]}\\ \end{aligned}$$

For the planar Laplace mechanism, the probability of perturbing the real location to different locations is the same:

$$\begin{aligned} Pr[PL(x^{'})=x^{'}]=Pr[PL(x_{i})=x_{i}] \end{aligned}$$

so that:

$$\begin{aligned} \frac{Pr[PL(x_{0})=x^{'}]}{Pr[PL(x_{0})=x_{i}]}&\le \frac{e^{\varepsilon \cdot d(x_{0},x^{'})}}{e^{\varepsilon \cdot d(x_{0},x_{i})}}\\ \end{aligned}$$

implying:

$$\begin{aligned} \frac{Pr[PL(x_{0})=x^{'}]}{Pr[PL(x_{0})=x_{i}]}&\le e^{\varepsilon \cdot (d(x_{0},x^{'})-d(x_{0},x_{i}))} \end{aligned}$$

In the triangle constructed by location points $x_{0}$, $x_{i}$, and $x^{'}$, the sum of the lengths of the two sides is always longer than the third one. Therefore, we have $d(x_{0},x^{'})-d(x_{0},x_{i})<d(x^{'},x_{i})$, so that:

$$\begin{aligned} \frac{Pr[PL(x_{0})=x^{'}]}{Pr[PL(x_{0})=x_{i}]} \le e^{\varepsilon \cdot (d(x^{'},x_{i}))} \end{aligned}$$

Therefore, for any perturbed location within the perturbation area $P_{area}$, the proposed Algorithm 1 can provide $\varepsilon$ geo-indistinguishbility protection for users’ location.

The proposed Algorithm 2 sets up the lower limitation of the number of users $\rho$. Therefore, the perturbed locations have the same semantic but the number of users less than $\rho$ will be excluded from $Q_{area}$. This will provide the privacy protection effect similar to the location *K*-anonymity. For attackers who can obtain the number and distribution of users, the proposed method will stop them from identifying specific users based on the outputs. Meanwhile, the proposed Algorithm 2 manages to delete the perturbed locations possessing the same or high similarity semantics as to that of the real ones. For attackers who want to infer users’ location and other privacy by comparing the prior and the posterior distribution of location semantics, the proposed perturbation method will not increase the attackers’ knowledge by observing the output results.

Combined with the above analysis, the location perturbation and optimization algorithm proposed in this paper can provide localized privacy protection for users’ location and resist the semantic related inference attack at the same time.

## Experimental results

In order to evaluate and analyze the location perturbation and optimization algorithm (marked as POLS) proposed in this paper, we compare it with a number of classical perturbation mechanisms from the aspects of LBS service quality loss, privacy protection strength, and range counting query accuracy. The baseline methods include, but are not limited to, local differential privacy perturbation mechanism (marked as KRR)^19^, geo-indistinguishability-based planar Laplace perturbation mechanism (marked as PL)^10^, geometric perturbation mechanism (marked as GEOM)^22^, and exponential perturbation mechanism (marked as EM)^23^.

**Figure 6:**
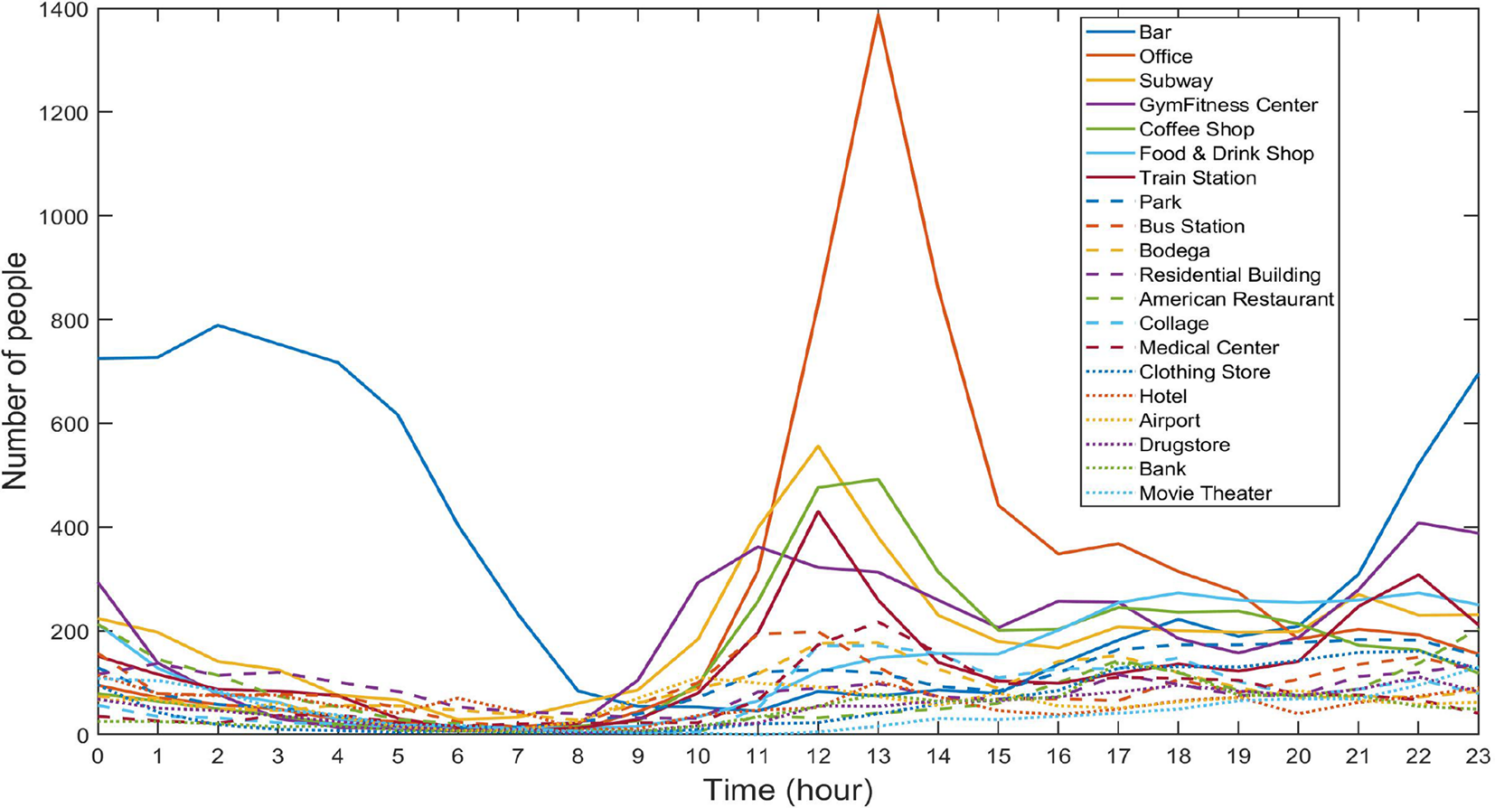
Temporal statistical properties of location semantics in the experimental dataset.

All the algorithms were programmed by MATLAB R2021a software and carried out in a hardware environment with AMD Ryzen 7 4800H at 2.90 GHz, 16GB memory, and Microsoft Windows 10 operating system. Use *Groubi* to perform the linear programming operations. The dataset used for the experiments includes 573,703 pieces of check-in information in Tokyo, Japan^49^ from April 12, 2012 to February 16, 2013. Each piece of the check-in information contains GPS coordinates, timestamp, and location semantics, which is used to study the spatio-temporal regularity of users’ activities in LBS system. We select twenty different types of location semantics from this dataset and portray the temporal statistical properties of these location semantics in Fig. 6. As can be observed from the Fig. 6, the places of entertainment, such as a bar, often meets peak business hours from late night to early morning. However, offices, subway stations, fitness centers, and coffee shops are busy during the working hours. The temporal statistical properties of location semantics in the experimental dataset is consistent with our ordinary experiences.

### Parameter configurations

We randomly select three sets of location points with scales of 50,000, 100,000, and 500,000 from the experimental dataset as the users’ real locations. As it can be observed from Fig. 6, the distribution characteristics of the same location semantic at different times are significantly different. Therefore, we implement three groups of experiments at 03:00, 12:00, and 18:00 respectively. During the experiments, the lower limitation of the number of users is $\rho =30$, and privacy parameter $\varepsilon \in \lbrace 0.004, 0.005, 0.007, 0.01, 0.02\rbrace$.

### Comparison of service quality loss

The local location perturbation mechanism generates a fake location to replace the user’s real location, therefore, the distance between the perturbed location and the real location can be used to intuitively reflect the quality loss of location-based services. In this paper, the mean value and variance of the distance between the perturbed location and the real location are used to measure the loss of location-based service quality caused by different perturbation mechanisms. The definitions are depicted in the following Eqs. (18) and (19).

18 $$\begin{aligned}{} & {} M_{dis}=\frac{1}{N}\times \sum _{i=1}^{N}dis(x_{i},x_{i}^{'}) \end{aligned}$$

19 $$\begin{aligned}{} & {} V_{dis}=\frac{1}{N}\times \sum _{i=1}^{N}(dis_{i}-M_{dis})^{2} \end{aligned}$$

**Figure 7:**
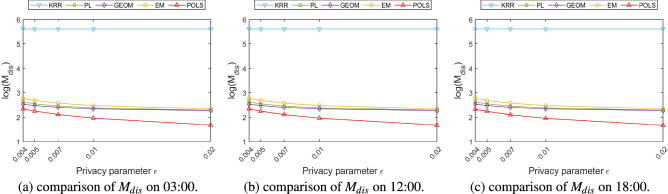
Comparison of $M_{dis}$ among different perturbation algorithms.

**Figure 8:**
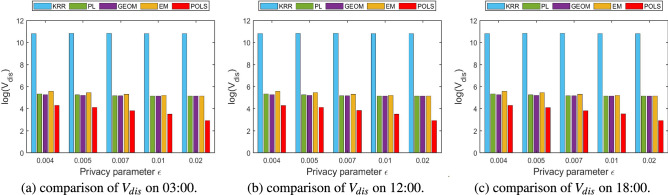
Comparison of $V_{dis}$ among different perturbation algorithms.

Figure 7 compares the mean of distances generated by different perturbation mechanisms under various privacy parameters over different time periods using the logarithmic coordinates. Figure 8 compares the variance of the distance between the perturbed location and the real location, which can facilitate comparing the degree of variation in the perturbed distance produced by different perturbation mechanisms. Comparing the mean and variance results of perturbed distances on different time periods, it can be observed that the perturbation results of various methods are not sensitive to time. Theoretically, the error and fluctuation range of the localized location perturbation mechanism is only related to the privacy parameters selected by the user but has nothing to do with the time when the perturbation operation is performed. From the actual experimental results, the mean distance error of various methods at different times is about 1 m. The error of KRR method is obviously higher than the other methods. In order to compare all the results together in one figure, we adopted the logarithmic coordinates to display the error results. Since most of the error results are in the same order of magnitude, such errors are less obvious in the logarithmic coordinate system. Detailed analysis of the above results show that the mean value and variance of the distance between the perturbed location and the real location generated by the KRR algorithm are significantly higher than those of other algorithms and is hardly varies with the change of privacy parameters. The main reason is that the KRR algorithm performs random response on user’s real location directly according to the local differential privacy model. All the location points in the entire geographic space have the same probability to be selected as perturbed location. The change of the privacy parameter $\varepsilon$ will only affect the probability that the user’s real location be selected to be the perturbed location, but will not make significant changes on the distance between the perturbed location and the real one. If the random response adopted by the KRR algorithm occurs on the higher bits of the latitude and longitude of users’ location, the deviation of the disturbed location from the real location will be large, resulting in a surge of loss of quality for location-based services.

Combining the results in Figs. 7 and 8 we can observe that the mean value and the variance of the distance generated by PL, GEOM, and EM mechanism are relatively close, and they all gradually decrease with the increase of the privacy parameter $\varepsilon$. The perturbation probability function of the GEOM mechanism is expressed in Eq. (20), wherein, $\lambda _{G}$ is a normalization parameter.

20 $$\begin{aligned} Pr[M_{G}(x_{0})=x]=\lambda _{G}\cdot e^{-\varepsilon \cdot dis(x_{0},x)} \end{aligned}$$

For the normalization of discrete probability functions, assuming that the entire space has a number of $N_{l}$ location points and the real location of the user is $x_{0}$, Eq. (21) can be used to obtain the normalization parameter $\lambda _{G}$.

21 $$\begin{aligned} \lambda _{G}=\frac{1}{\sum ^{N_{l}}_{i=1}e^{-\varepsilon \cdot dis(x_{0},x_{i})}} \end{aligned}$$

The perturbation probability function of the EM mechanism can be expressed as Eq. (22):

22 $$\begin{aligned} Pr[M_{E}(x_{0})=x]=\frac{e^{-\frac{\varepsilon }{2}\cdot dis(x_{0},x)}}{\sum ^{N_{l}}_{i=1}e^{-\frac{\varepsilon }{2}\cdot dis(x_{0},x)}} \end{aligned}$$

Comparing the perturbation probability function of the above three mechanisms, it can be found that the same privacy parameter $\varepsilon$ achieve different perturbation probabilities in the PL, GEOM, and EM algorithms and result in different perturbed distances. The same privacy parameter $\varepsilon$ obtains more amount of perturbations while using the EM algorithm, therefore, the corresponding perturbed distance is farther and the mean value and the variance of the distance are larger than others.

The proposed POLS algorithm obtains the radius of the perturbed area corresponding to certain privacy parameter $\varepsilon$ based on the geo-indistinguishability mechanism, and restricts all the possible perturbed locations within this area to limit the variation range of the mean value and variance of the perturbed distance. Table 2 depicts the radius of the perturbed area and it’s corresponding differential privacy parameter $\varepsilon$ generated by Algorithm 1. The radius of the perturbed area is gradually decreased with the increase of the privacy parameter $\varepsilon$. Therefore, the proposed POLS method received lower perturbation distance on various time periods. Compared with the PL, GEOM, and EM algorithms, the mean value and the variance of the proposed POLS method has reduced about 37%.

**Table 2:** Radius of perturbation area vs. privacy parameter $\varepsilon$.

${\varepsilon }$	Radius (m)
0.004	500
0.005	400
0.007	300
0.01	200
0.02	100

### Comparison and analysis of privacy protection degree

The attackers may intercept LBS requests submitted by users and infer additional privacy based on location information. The smaller the semantic correlation between the perturbed location generated by the local perturbation mechanism and the user’s real location, the less likely the attackers can infer the users’ privacy. Therefore, we use the cosine similarity between the perturbed location and the real location to evaluate the privacy protection degree of different perturbation mechanisms. The calculation method of cosine similarity is defined in Eq. (11).

**Figure 9:**
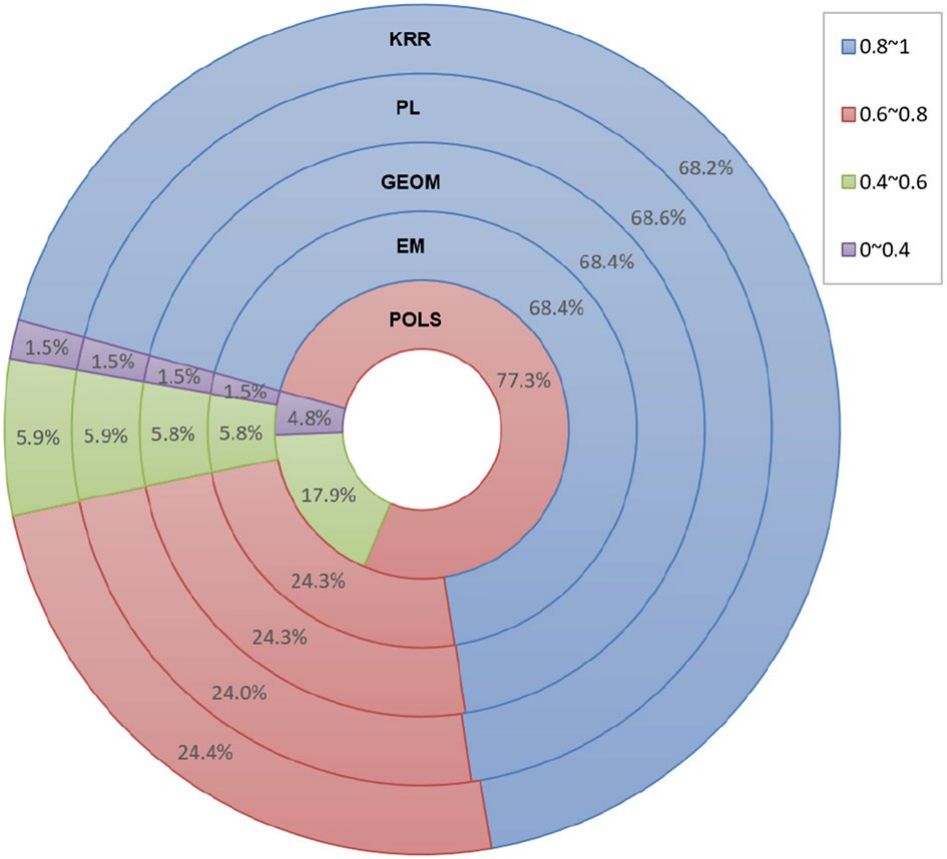
Distribution ratio of cosine similarity.

**Figure 10:**
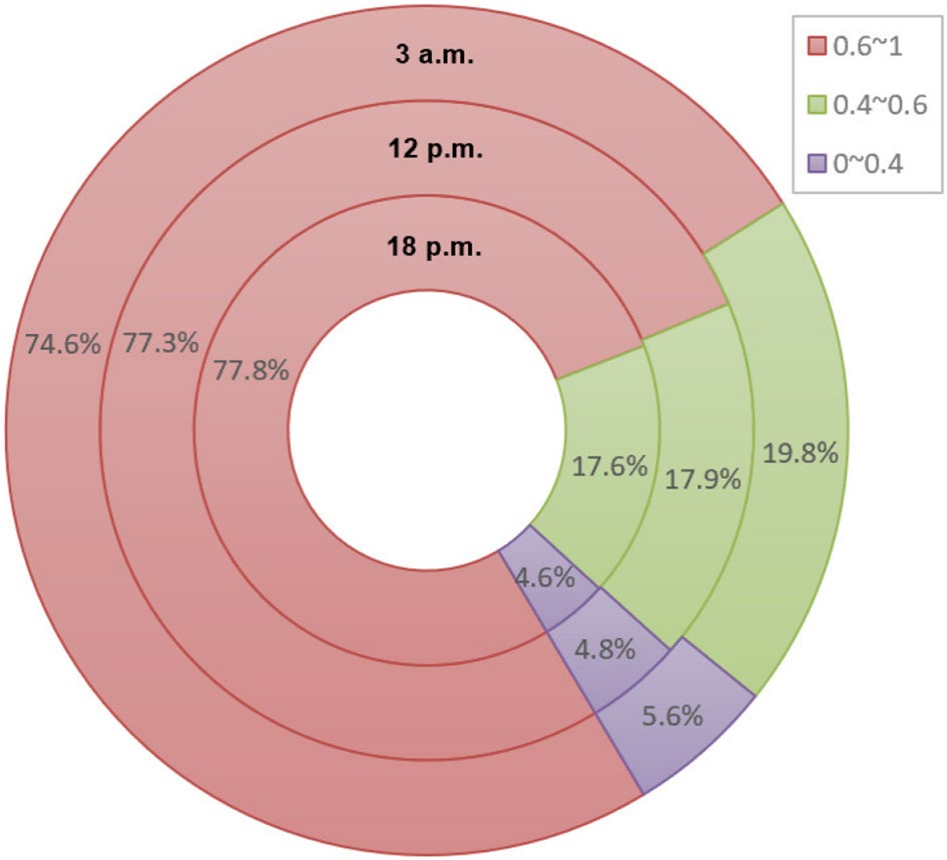
Cosine similarity of POLS algorithm.

Figure 9 depicts the distribution ratio of the cosine similarity between the perturbed location and the real location generated by different perturbation mechanisms on the experimental dataset at 12:00 pm Since the setting of the privacy parameter has no effect on the distribution ratio of the cosine similarity, we only take $\varepsilon =0.02$ as an example for analysis. Each of the ring in Fig. 9 represents a perturbation mechanism and different colors stand for the distribution ratio of the cosine similarity between the perturbed location and the real location. It can be observed that in addition to the proposed POLS algorithm, the cosine similarity between the perturbed location and the real location generated by other algorithms is mainly distributed within the interval [0.8, 1]. As mentioned above, a higher cosine similarity means that the perturbed location has a higher semantic similarity with the real location. Although the attackers may not directly obtain the users’ precise location, they can analyze the users’ behaviors, hobbies, habits, and many other privacy information according to the location semantics. On the contrary, the cosine similarity of the proposed POLS algorithm is mainly distributed within the interval [0.6, 0.8]. The proportion of the cosine similarity less than 0.6 reaches $22.7\%$, which is much higher than the level about $7\%$ for other algorithms. The results proved that the perturbed location generated by the proposed POLS algorithm has lower semantic similarity with the real location, which facilitate to resist semantic inference attacks and provide users with better location privacy protection.

Figure 10 further compares the distribution ratio of the cosine similarity between the perturbed location and the real location generated by the proposed POLS algorithm in different time periods under the premise of the same privacy parameter ($\varepsilon =0.02$). Although the number of users distributed on different semantic locations at different times is quite different, the proposed POLS algorithm can overcome the temporal difference of semantic location distribution and provide more consistent perturbation location generation effects in different time periods.

### Comparison and analysis of range counting query accuracy

Location-based big data services collect and organize location information from various terminals and channels, and provide users with services such as inquiry of points of interest within a certain range, the number of other users, the number of available vehicles, traffic conditions, etc. In this section, the accuracy of the range counting query service is used to measure the availability of perturbed location data submitted by users. For the query range submitted by the users, the relative error between the real location dataset and the perturbed location dataset can be calculated according to Eq. (23).

23 $$\begin{aligned} RE(Q)=\frac{|C^{*}(Q)-C(Q)|}{max\{C(Q),\beta \}} \end{aligned}$$

wherein, *Q* represents the query range submitted by the user, *C*(*Q*) is the statistical result within the query range obtained on the real location dataset, and $C^{*}(Q)$ is the statistical result within the query range obtained on the perturbed location dataset. To prevent the denominator from being zero, we set $\beta =0.001\times |T|$, wherein |*T*| represents the size of the experimental location dataset.

During the experiments, we randomly generated three different scales location datasets within the area of city Tokyo at 12:00 pm, when the users’ activity patterns were the most abundant. The corresponding perturbed location datasets are obtained by performing different perturbation algorithms on the three original location datasets mentioned above. Three sizes of spatial query ranges are set, which cover 5%, 15%, and 45% of the spatial area of the real location dataset respectively. Each of the query was randomly selected and executed for 10,000 times to determine the average relative error.

**Figure 11:**
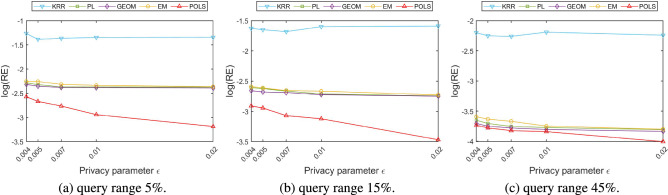
Comparison of range counting query accuracy (dataset with 50,000 users).

**Figure 12:**
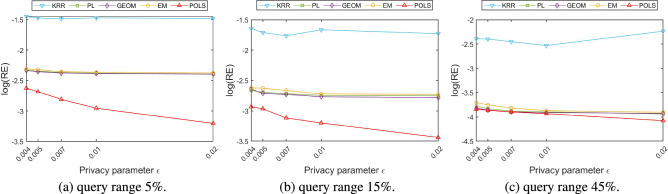
Comparison of range counting query accuracy (dataset with 100,000 users).

**Figure 13:**
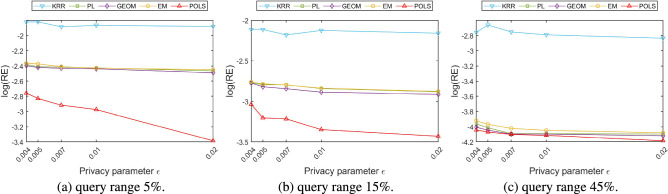
Comparison of range counting query accuracy (dataset with 500,000 users).

Figures 11, 12 and 13 portray the relative error comparison results of various algorithms on different datasets in logarithmic scale. From the macro comparison of three location datasets of different sizes, the relative error of the range counting query gradually reduced with the increase of the number of users. The reason is that when the overall number of users is small, the distribution is relatively sparse and the change of users’ location may lead to large deviations in the statistical results in a local area. As the overall number of users increases, the distribution density is also increases. The location change of some users takes them out of their original local area, while the location change of other users may bring them into this local area. Therefore, this kind of mutual cancellation reduces the bias of the range counting statistics. On the same location data set, the relative error is also decreased as the query range increases from small area to large area. The main reason is that when the query range is small, some local users leave the current range after the location perturbation, resulting in a high relative error of the range counting query. With the increase of the query range, the perturbation results of users’ location may deviate from their original area, but it seldom exceed the query range, therefore, the relative error of the range counting query is also reduced.

When we compare the relative error of various location perturbation algorithms under the same dataset and query range, it can be observed that the relative error of the KRR algorithm does not change significantly with the change of the privacy parameters $\varepsilon$. Since the random response technology adopted by KRR algorithm is not directly related to the degree of location perturbation and the change of privacy parameter. The relative errors of the other algorithms gradually decrease with the increase of the privacy parameter $\varepsilon$. The reason is that the increase of the privacy parameter $\varepsilon$ will reduce the incorporated perturbation value, so that the error between the published location and the real location also decreased. The location perturbation and optimization algorithm proposed in this paper aims at minimizing the quality loss of location-based services. The constructed service quality loss function comprehensively considers the distance between the perturbed location and the real location as well as the prior probability of users distribution. The above factors facilitate to constrain the users’ perturbed location within a reasonable range. Therefore, the proposed location perturbation and optimization algorithm achieves better query accuracy than the other algorithms on datasets of various scales and with different privacy parameters. Taking the distribution of the most sparse number of users as an example, when the querying range is 5%, the relative error of the proposed algorithm is reduced about 43% in contrast to the other methods; when the querying range is 15%, the relative error of the proposed algorithm reduces about 44% than the others; and when the query range is 45%, the relative error of the proposed algorithm is reduced about 5% in contrast to the other methods.

## Conclusions

Popular application fields of big data such as Internet of things, intelligent transportation, location-based services and mobile crowd-sensing are collecting and using users’ location information all the time. While bringing unprecedented convenience to users, the protection of location privacy has also attracted extensive attentions. Localized perturbation mechanism allows users to protect their locations according to personal requirements which breaks the dependence on the trusted third-party platforms and provide stronger privacy protection for end users. This paper proposes a location perturbation and optimization method for terminal users, which generates the perturbed location area conforms to geo-indistinguishability according to the plane Laplace mechanism, optimizes the perturbed location area using the average similarity of location semantics, and selects the optimal perturbed location by the linear programming method. The proposed method not only achieves location privacy via geo-indistinguishability model but also protects the sensitivity of the location through location semantics. Therefore, it can protect users’ trajectory and avoid the semantic correlated inference of the adversary in the long term.

However, the research still has some limitations. Firstly, the motivation of this work is to protect the exact locations of users and maintain the data availability while using the location-based services. The proposed perturbation and optimization method can provide location privacy for a single request of LBS or solve the location protection problems by discretizing the continuous query into a finite set of single queries. For users who need to perform LBS queries continuously on spatiotemporal correlated locations, it is necessary to improve the proposed method and generate continuous policy of privacy budget allocation and perturbation scheme. Secondly, owing to the sematic information of the experimental dataset, the proposed method combines the quantity of people with a certain kind of location semantic and it’s respective time characteristics to get the average similarity of location semantics. Some other available types of location semantic information such as the relationships between locations, the number of visits to a location, the durations of the visits, and the distances users travel to reach locations can be employed to improved the effect of location privacy. How to refine the experimental datasets with the above location semantic information will be one of the future directions of this paper. Finally, as we discussed in the related work, although the LDP-based location perturbation method provides stronger guarantees of privacy compared with the centralized DP model, the aggregator on the server side will achieve more statistical errors than the DP-based methods. Some researches suggest to combine the LDP perturbation method with the shuffled model so as to obtain accurate statistics while keeping raw data in users’ hands. This also provides a feasible direction for the further improvement of our proposed method.

## Data Availability

All data generated and analysed during this study are included in this published article^49^.
